# Childhood severe asthma: relationship among asthma control scores, FeNO, spirometry and impulse oscillometry

**DOI:** 10.1186/s12890-024-03058-x

**Published:** 2024-06-06

**Authors:** Gabriela Peláez, Verónica Giubergia, Belén Lucero, Verónica Aguerre, Claudio Castaños, Juan Manuel Figueroa

**Affiliations:** 1 Pulmonology Department, Hospital de Pediatria Dr. Juan P. Garrahan, Combate de los Pozos 1881 (1245), Buenos Aires, Argentina; 2 Pediatric Pulmonology Section, Hospital de Clínicas “José de San Martín”, Buenos Aires, Argentina; 3 Fundación Pablo Cassará, Buenos Aires, Argentina

**Keywords:** Clinical scores, Children, Nitric oxide, Pulmonary function test, Severe asthma

## Abstract

**Introduction:**

The evaluation of the asthmatic patient is usually based on clinical and functional parameters that do not necessarily evidence the degree of airway inflammation. The aim of this study was to analyze whether clinical scores (CS) correlate with spirometry (S), impulse oscillometry (IO) and FeNO, in severe asthmatic children.

**Material and methods:**

A multicentric, prospective, cross-sectional study was conducted over a 12-month period. All SA patients (6–18 years old) followed-up in the Pulmonology Department were recruited. CS, FeNO measurements, IO and S were consecutively performed on the same day. Asthma control was ascertained using ACT and GINAq. A cut-off value of ≥ 25 parts per billion (ppb) was used to define airway inflammation.

**Results:**

Eighty-one patients were included. ACT: 75% (n 61) were controlled; GINAq: 44.5% (n 36) were controlled; 39.5% (n 32) were partly controlled, and 16% (n 13) were uncontrolled. FeNO had a median value of 24 ppb (IQR 14–41); FeNO ≥ 25 ppb was observed in 49% of patients (n 39). ROC AUC for FeNO vs. ACT was 0.71 (95%CI 0.57–0.86), PPV 0.47, NPV 0.87, SE 0.61, SP 0.80; FeNO vs. GINAq was ROC AUC 0.69 (95%CI 0.54–0.85), PPV 0.34, NPV 0.91, SE 0.62, SP 0.77; Youden cut-off FeNO > 39 ppb for both CS.

**Conclusion:**

In severe asthmatic children, current symptoms control as evidenced by ACT and GINA correlates with low FeNO values. Clinical scores showed good correlation with airway inflammation.

## Introduction

Asthma is the most common chronic respiratory disease worldwide affecting an estimated 262 million people in 2019 and caused 455.000 deaths [1].

Up to 10% of adults and 2.5% of children with asthma have severe asthma (SA) [2]. Children with SA have uncontrolled asthma despite adherence with maximal optimized high-dose inhaled glucocorticoids (ICS) and long-acting β2-agonits (LABA) treatment and management of contributory factors, or that worsen when high-dose treatment is decreased [3].

In Argentina, asthma accounts for more than 400 annual deaths (10% in patients aged 5 to 39 years) and more than 15.000 hospitalizations per year, especially in patients with more severe disease [4].

Achieving adequate control is the final objective in the follow-up of asthmatics, regardless of the severity of the disease. Asthma Control Test (ACT) and GINA Asthma Control Questionnaire (GINAq) are validated clinical scores (CS) widely used to assess the degree of disease control based on clinical criteria [3].Pulmonary function has also been proposed as a measure to evaluate asthma control, although in pediatrics, the evidence is scarce [5].

Inflammation parameters are not considered in the evaluation of asthma control by CS, whereas chronic airway inflammation is the hallmark of asthma. Nitric oxide (NO) is an important regulator of immune responses and is a product of inflammation in the airways that is over-produced in asthma. Fractional exhaled nitric oxide (FeNO), a non-invasive method, allows indirect evaluation of type 2 airway inflammation [6].

Asthma control is a multidimensional measure with features that are complementary to each other, including clinical, functional and disease activity. Hence, a quick and easy assessment may not offer a comprehensive or precise estimation of asthma control. CS are accessible and easy tools to evaluate the degree of asthma control in daily practice, while FeNO and pulmonary function test (PFT) equipment are not always available in public health services, due to high cost. Currently, the evaluation of pulmonary function and airway inflammation together with asthma control has been scarcely studied in general, particularly in SA children.

The aim of the study was to analyze whether ACT and GINAq correlates with spirometry (S), impulse oscillometry (IO) and FeNO. The hypothetical agreement among them would be very useful in centers where PFT and FeNO equipment are not available for SA children follow-up.

## Materials and methods

A multicentric, prospective, cross-sectional study was conducted over a 12 months period. All SA patients (according to GINA guidelines), aged 6–18 years, with ≥ 12 months of diagnosis, followed-up in the SA Program at “Hospital de Pediatría Garrahan” (n 60) and “Hospital de Clínicas Jose de San Martín” (n 26), were consecutively recruited (n 86) [3, 4, 7].

CS, FeNO measurements, IO and S were consecutively performed on the same day. The health care professional that performed FeNO, IO and S was blind to CS results. Asthma control was ascertained using ACT and GINAq. ACT scores of ≥ 20 means well-controlled asthma [3, 8]. GINAq characterizes asthma control in three levels: “controlled”, “partly controlled” and “uncontrolled” [3].

Mean FeNO values out of two measurements (variability ≤ 10%), were recorded [9]. A cut off value of ≥ 25 parts per billion (ppb) was used to define airway inflammation [10].

As intra-individual FeNO levels vary across devices, all measurements were performed with the same NoBreath equipment (Bedfond Ltd, United Kingdom). Forced vital capacity (FVC), forced expiratory volume in 1 s (FEV_1_), FEV_1_/FVC ratio, forced expiratory flows between 25 and 75% of FVC (FEF_25–75_) were analyzed. All parameters were expressed as percentage (%) of the predictive value. Bronchodilator response (BDR) was evaluated 15 min after administration of 400ug of salbutamol through a spacer device. Patients were instructed to withdraw salbutamol 4 h and LABA 12 h before tests. A significant BDR was considered a 12% and 200 ml increase of initial FEV_1_ [3, 11–13].Jaeger Master Screen equipment was used.

Impedance 5 Hz (Z5), resistance 5 Hz, 10 Hz and 20 Hz (R5, R10, R20), reactance 5 Hz (X5), resonance frequency (Fres) and the area under the curve (AX) of the respiratory system were registered. The average values of at least three maneuvers with consistency > 0.6 at 5 Hz and > 0.9 at 10 Hz and coefficient of variation (CV) < 10%, were registered [14, 15]. Bronchodilator response (BD) was defined as a decrease of ≥ 40% in R5 and/or ≥ 80% decrease in AX and/or an increase of 50% in X5 [16]. FENO and PFT were performed following ATS/ERS recommendations [9, 11–16].

Those children with respiratory infection or asthma exacerbation were rescheduled. Patients with inability to perform PFT/FeNO maneuvers or who refused to sign the informed consent were excluded. All parents signed a written informed consent. The study was approved by the Garrahan´s Hospital Ethics Committee (Ref Proj 1022).

### Statistic analysis

Continuous data were summarized by the arithmetic mean and standard deviation or 95% confidence interval. To compare ACT and GINAq categories, Student test, Mann-Whitney test and Chi2 test were applied as appropriate. For a better definition of uncontrolled cases GINAq categories were grouped as uncontrolled versus controlled and partially controlled asthma.

To evaluate CS vs. FeNO and PFT performance, a receiver operating characteristic (ROC AUC), Youden cut-off and positive predictive value (PPV) /negative predictive value (NPV) were applied. A p value < 0.05 was considered statistically significant. Stata XIV software was used (Stata-Corp, College Station, TX).

## Results

Eightysix cases were recruited. Five patients were excluded due to inability to perform PFT (n 1) or missed visits (n 4). Considering ACT, 75% of children (n 61) were controlled. According to GINAq, 44.5% (n 36) were controlled, 39.5% (n 32) partly controlled and 16% (n 13) uncontrolled. Characteristics of the population are shown in Table 1.

**Table 1:** Characteristics of study population (n 81)

Characteristic		
Age (years old)	Median (IQR)	12 (9–14)
Male Sex	% (n)	46 (37)
ICS†	Median (IQR)	800 (520–1240)
Leukotriene receptor antagonists	% (n)	41 (33)
Oral corticosteroids	% (n)	5 (4)
Omalizumab	% (n)	20 (16)
BMI	Median (IQR)	22 (19–26)
Obesity	% (n)	62 (50)
Blood Eosinophils	Median (IQR)	489 (240–682)
Rhinitis	% (n)	74 (60)
Eczema	% (n)	23 (19)
OSA	% (n)	11 (9)
ACT	% (n)	
Controlled Uncontrolled		75 (61) 25 (20)
GINA	% (n)	
Controlled/Partly controlled Uncontrolled		84 (68) 16 (13)
FeNO (ppb)	Median (IQR)	24 (14–41)
**Spirometry**	Mean (SD)	
FVC		112 (15)
FEV_1_		104 (17)
FEV_1_/FVC		81 (9)
FEF _25−75_		86 (31)
**IO**	Median (IQR)	
Z5Hz		89 (75–103)
R5Hz		89 (73–103)
R10Hz		88 (75–104)
R20Hz		88 (75–104)
X5Hz		96 (79–123)

†Inhaled corticosteroids (ICS), ug: Budesonide or equivalent

BMI: Body Mass Index

ACT: Asthma Control Test

OSA: Obstructive Sleep Apnea

Reliable values of FeNO were obtained in 97.5% of cases (n 79), with a median value of 24 ppb (IQR 14–41). FeNO ≥ 25 ppb was observed in 49% (n 39) of them (median 41 ppb; IQR 33–97), irrespective of asthma control.

Subjects with uncontrolled asthma by ACT had significantly higher FeNO than controlled ones: 42 ppb (IQR 28–89) vs. 20 ppb (IQR 13–36) (p 0.006). FeNO was also high in GINAq uncontrolled vs. controlled and partly controlled cases: 41 ppb (IQR 28–89) vs. 21 ppb, (IQR 13–37) respectively (p 0.02). Tables 2 and 3.

**Table 2:** FeNO and pulmonary function test according to ACT values

	ACT ≤ 19 (*n* 20) Uncontrolled	ACT > 19 (*n* 61) Controlled	*P*
**FeNO**(ppb)†	42 (28–89)	20 (13–36)	**0.006**
**Spirometry**‡			
FVC	112 (12)	112 (16)	0.48
FEV_1_	102 (14)	104 (17)	0.72
VEF_1_/FVC	79 (10)	81 (9)	0.79
FEF _25−75_	78 (24)	89 (32)	0.89
**IO**†			
Z5Hz	91 (75–118)	89 (72–100)	0.37
R5Hz	91 (73–110)	87 (72–100)	0.31
R10Hz	86 (74–116)	89 (75–102)	0.63
X5Hz	104(76–123)	92 (79–122)	0.53
BDR § ¶	6 (30)	10 (16)	0.18

†Median (IQR),‡ Mean (SD),§ BDR in IOS and/or spirometry, ¶ (n, %)

FeNO: Fractional exhaled nitric oxide ACT: Asthma Control Test BDR: Bronchodilator response

**Table 3:** FeNO and pulmonary function test according to GINA questionnaire

	GINAq (*n* 13) Uncontrolled	GINAq (*n* 68) Controlled – partially controlled	*P*
**FeNO** (ppb)†	41 (28–89)	21 (13–37)	**0.02**
**Spirometry‡**			
FVC	112 (15)	112 (15)	0.51
FEV_1_	103 (18)	104 (17)	0.59
VEF_1_/FVC	80 (12)	81 (9)	0.59
FEF _25−75_	80 (30)	87 (31)	0.73
**IO†**			
Z5Hz	87 (75–118)	89 (72–101)	0.71
R5Hz	86 (75–109)	90 (72–100)	0.64
R10Hz	85 (74–104)	90 (75–103)	0.80
X5Hz	100(76–121)	96 (79–123)	0.96
BDR § ¶	4 (31)	12 (18)	0.27

†Median (IQR), ‡Mean (SD), § BDR in IOS and/or spirometry, ¶ (n, %)

FeNO: Fractional exhaled nitric oxide. GINAq: GINA Asthma Control Questionnaire. BDR: Bronchodilator response

FeNO values ≥ 25 ppb was observed in 70% (n 14) of uncontrolled cases by ACT (median 63 ppb, IQR 39–97), and in 77% (n 10) of GINAq uncontrolled ones (median 55 ppb, IQR 39–97). Table 4.

**Table 4:** FeNO measurement according to asthma control by ACT and GINAq

	ACT ≤ 19 (*n* 18)	ACT > 19 (*n* 61)	GINAq NC (*n* 13)	GINAq C-PC (*n* 66)
**FeNO < 25 ppb**				
% (n)	20 (4)	59 (36)	23 (3)	54 (37)
Median (IQR)	12 (8–14)	14 (11–19)	14 (8–23)	14 (11–19)
**FeNO ≥25 ppb**				
% (n)	70 (14)	41 (25)	77 (10)	43 (29)
Median (IQR)	63 (39–97)	38 (33–90)	55 (39–97)	39 (33–90)

FeNO: Fractional exhaled nitric oxide ACT: Asthma control test GINAq: GINA Asthma Control Questionnaire

**Fig. 1:**
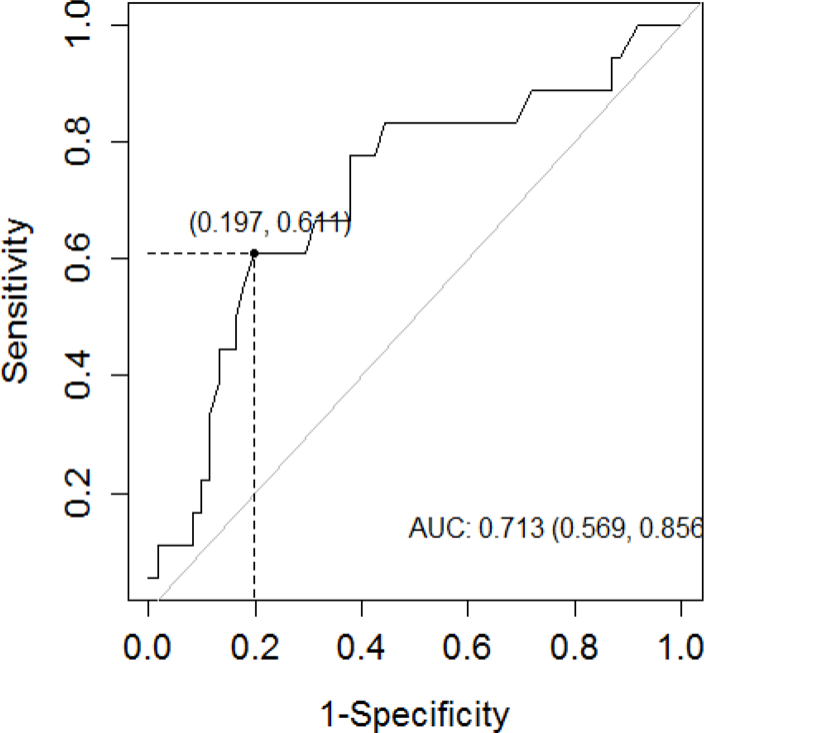
Receiver operating characteristic (ROC) curve analyses of FeNO values to determinate asthma control following ACT, AUC = 71%

**Fig. 2:**
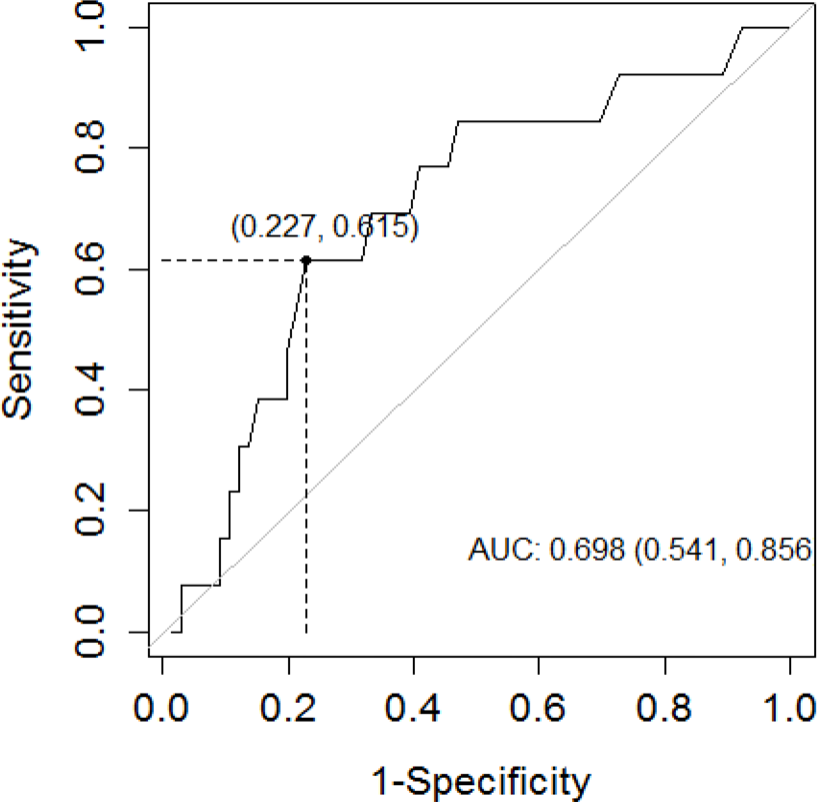
Receiver operating characteristics (ROC) curve analyses of FeNO values to determinate asthma control following GINAq, AUC = 69%

A ROC curve was generated to predict the identification of uncontrolled individuals using the measurement of FeNO and PFT. On comparing the sensitivity (SE), specificity (SP), PPV and NPV, and AUC ROC curve, the best combination without a significant loss of SE was a FeNO level > 39 ppb for both ACT and GINAq (Youden cutoff). The ROC AUC for FeNO vs. ACT was 0.71 (95%CI 0.57–0.86), PPV 0.47, NPV 0.87, SE 0.61, SP 0.80 (Fig. 1); FeNO vs. GINAq was ROC AUC 0.69 (95%CI 0.54–0.85), PPV 0.34, NPV 0.91, SE 0.62, SP 0.77 (Fig. 2). Patients with low FeNO had an 87% and 91% of probability of being controlled according to ACT and GINAq, respectively.

Spirometry was performed in 94% (n 76) of cases; reversibility was observed in 21% (n 16) of them; 56.6% (n 43) of patients evidenced mild obstruction, 1 moderate and 1 severe airway obstruction. All patients performed IOS. Normal values were observed in 92.5% (n 75), 2% (n 2) evidenced reversibility.

In uncontrolled cases, according to S, mild obstruction was observed in 70% (n 14) and 62% (n 8) by ACT and GINAq respectively. Considering IO, 20% (n 4) and 23% (n 3) evidenced pathological values by ACT and GINAq accordingly.

There was no significant association between the degree of asthma control neither by ACT nor GINAq when PFT was analyzed. No correlation was observed when ROC AUC was applied. Tables 2 and 3.

## Discussion

The results of the present study, which aimed to determine the agreement between asthma control defined by GINA questionnaire and ACT, airway inflammation and pulmonary function, showed two main findings. First, FeNO values but not lung function (spirometry/IO) was shown to correlate with asthma control. Second, patients with low FeNO had up to 91% of probability of being controlled according to CS.

These results indicate a good correlation between current asthma symptom control and the control of airway inflammation, irrespective of pulmonary function.

Proper asthma control is the goal of asthma management worldwide. It is easily evaluated through clinical questionnaires. Several numeric scores have been developed for children like ACT, ACQ among others. ACT is widely used and their Spanish version has been validated [3, 8, 17]. Such patient-reported outcome measures are considered to be clinically relevant because they are strong predictors of future exacerbations [18]. The results of these tests correlate to some extent with each other and with GINA classification of symptom control [3]. In our series, 75% of children according to ACT, and 84% GINAq were controlled. These scores define control by a composite measure of clinical findings but without using markers of airway inflammation, the hallmark of asthma [19].

In recent years, the study of airway inflammation has gained relevance for asthmatics follow-up [19]. Measurement of the FeNO is an easy technique to use, provides immediate results, is noninvasive, and is a reproducible biomarker of airway inflammation in asthma. However, the high costs of the equipment still hinder its wide use in public health services, especially in countries like Argentina, with limited health resources [20].

Although there is strong evidence that the levels of FeNO correlate with features of type 2 inflammation [21], its ability to predict asthma control has been evaluated with contradictory results [5, 10, 22–25].

Ricciardolo and colleagues verify whether the FeNO measurement could be associated with clinical and functional factors for the evaluation of asthmatic patients in a real-life situation. FeNO was associated with uncontrolled asthma at the cut-off point of FeNO > 29.95 ppb and an area under the ROC curve of 0.70 [25]. de Abreu and colleagues found that FeNO level could be helpful in determining asthma control as > 30 ppb was associated with uncontrolled asthma [24].These values were close to the cut point of 39 ppb of our study. Other authors who evaluated the association between the FeNO and asthma control, based on the GINA criteria, found no statistically significant difference [22, 23].

Discrepancies could be explained by the inclusion of different groups of individuals. It is worth mentioning that studies included mainly mild and moderate adults asthmatic patients [24, 25].

Our results are in keeping with previous reports showing that the ability of clinical assessment to predict the presence and type of inflammation was good [24, 26].

The present study, conducted in a well characterized population of SA children has shown a significant correlation between the degree of asthma control and FeNO values with high negative predictive values for both clinical scores. According to ACT and GINAq individuals with low FeNO had an 87% and 91% of probability of being controlled respectively.

Negative responses to the four questions of the GINAq and ACT are good indicators of the control of airway inflammation. In contrast, it was not possible to confirm otherwise. The presence of symptoms was not an indicator of airway inflammation. Due to the low PPV observed in our study, it cannot be inferred that FeNO could be elevated in uncontrolled cases. However, a median FeNO of 41–42 ppb was observed in uncontrolled ones. Accordingly, 20–23% of children with uncontrolled asthma had low FeNO, suggesting that in these scenarios, other underlying physiopathologies or causes may explain the symptoms.

It has been difficult to provide exact FeNO cut-off values for clinicians due to heterogeneity of values used across studies. In children, FeNO cut-points are slightly different.

For clinical practice, ERS and ATS consider that FeNO between 20 and 35 ppb should be judged within the clinical context and values > 35 ppb may be used to indicate that type 2 inflammation is likely [19]. These values are very close to 39 ppb found in our study. The question arises as to whether well or totally controlled asthma based on clinical criteria alone, reflects an adequate control of the underlying airway inflammation. While FeNO > 25 ppb may be abnormal in healthy subjects, in patients with well-controlled asthma, such a value is common, and a growing body of evidence suggests that cut-offs should be based on characteristics of the population of interest [27]. In our population, children with well-controlled asthma had a median FeNO level of 20–21 ppb.

ROC showed that 39 ppb was the best cut-point based on the SE and SP for both scores.

The data of this study suggest that lung function is an inadequate tool for predicting asthma control, in agreement with other reports [24]. Of uncontrolled cases, 15% and 80% had shown normal S or IOS values, respectively and up to 70% evidenced mild obstruction. The normal or almost normal baseline values observed in our series reveal the lack of sensitivity of the PFT to correlate with the degree of symptom control. It is striking that 62–70% of uncontrolled patients with an almost normal baseline spirometry, remain without adequate asthma control. A hypothesis that could explain the slight changes observed in the pulmonary functions in patients with frequent symptoms would be given by the increased bronchomotor tone and its lability [4, 28, 29].

Recently it has been shown the additive effects of combining spirometry with oscillometry in adults with moderate-to-severe asthma [30–32]. In adults, severe asthma is closely associated with major lung function changes, which are not observed in children, as previously described [33, 34]. Children with severe asthma tended to have less severe airflow obstruction compared to adults [33]. Spirometric measurements are insensitive discriminators of problematic severe asthma in childhood [34].

Hence, a discordant pattern of generally low correlations between measures of airway inflammation, clinical parameters, with pulmonary function, as shown in our study, may not be surprising.

The GINA guidelines reports that lung function is not strongly correlated with symptoms of asthma, suggesting the use of other instruments of control, and includes elevated FeNO as a predictor of exacerbations [3]. In children, pulmonary function measurement is a useful and very specific tool for asthma diagnosis, although not sensitive enough for follow-up of the most severe cases [4, 35].

Thus, our results reinforce the superiority of inflammatory markers over functional tests regarding asthma control. The level of airway obstruction is more related to risk of exacerbations than asthma control [3].

A limitation of our study might be the use of a specialized clinic sample in respiratory disease, which may have introduced a selection bias. Patients at specialized outpatient clinics tend to have more severe disease and do not represent patients with asthma evaluated by a general practitioner. All asthmatics were on long-term high doses of inhaled corticosteroids. Inclusion of a group of steroid free asthmatics would have facilitated the potential association of CS with airway inflammation and lung function parameters.

The data presented in this study demonstrate that in SA children current symptom control correlates with low FeNO suggesting that conventional asthma clinical measures like ACT and GINAq reflex control of airway inflammation. They are complementary tools. When FeNO equipment is not available, clinical scores might provide useful information for the follow-up of these patients.

## Data Availability

The datasets used and analyzed during the current study are available from the corresponding author on reasonable request.

## Abbreviations

ACT: Asthma control test
AX: Area under the curve
BDR: Bronchodilator response
CS: Clinical scores
CV: Coefficient of variation
FEF_25–75_: Forced expiratory flows between 25 and 75% of FVC
FeNO: Fractional exhaled nitric oxide
FEV_1_: Forced expiratory volume in 1 s
Fres: Resonance frequency
FVC: Forced vital capacity
GINAq: GINA asthma control questionnaire
IO: Impulse oscillometry
ICS: Inhaled glucocorticoids
LABA: Long-acting β2-agonits
NO: Nitric oxide
NPV: Negative predictive value
OSA: Obstructive Sleep Apnea
PFT: Pulmonary function test
PPV: Positive predictive value
R5: Resistance 5 Hz
R10: Resistance 10 Hz
ROC AUC: Receiver operating characteristic
S: Spirometry
SA: Severe asthmatic
SE: Sensitivity
SP: Specificity
X5: Reactance 5 Hz
Z5: Impedance 5 Hz
